# A Rare ST-Elevation Myocardial Infarction Mimic or a True Event?

**DOI:** 10.7759/cureus.7569

**Published:** 2020-04-07

**Authors:** Talha Ahmed, Melsjan Shkullaku

**Affiliations:** 1 Internal Medicine, University of Maryland Medical Center, Baltimore, USA; 2 Internal Medicine: Cardiology, University of Maryland Medical Center, Baltimore, USA

**Keywords:** st elevation mi, plaque rupture, plaque erosion, coronary angiography, acute coronary syndrome, electrocardiogram

## Abstract

The etiology of ST-elevation myocardial infarction (STEMI) is either rupture or erosion of unstable plaque with subsequent thrombosis. With the widespread use of plaque-stabilizing lipid-lowering therapies (statins), plaque erosion, rather than rupture, now accounts for most cases of acute coronary syndromes (ACS). In the spectrum of ACS, STEMI usually results from the total occlusion of the culprit epicardial coronary artery, leading to the occlusion of blood flow to the affected myocardium. The differential diagnosis of ST-elevations on electrocardiograms are broad. However, an elevated cardiac marker, evidence of wall motion abnormality on echocardiogram or positive stress testing makes an alternate diagnosis less likely. This prompts emergent coronary angiography with an intent to fix the underlying cause. In some cases like ours, when the clinical suspicion of STEMI is high, the coronary angiography may be unrevealing of the diagnosis.

## Introduction

ST-elevation myocardial infarction (STEMI) is usually caused by plaque rupture or erosion leading to the total occlusion of the coronary arteries. Myocardial infarction in the absence of critical epicardial coronary vessel disease is being increasingly recognized (10% of cases of acute myocardial infarction). However, STEMI usually involves some form of culprit lesion that might be either an unstable plaque with erosion or rupture with overlying thrombus formation leading to total occlusion of epicardial vessels [1-2]. We describe the case of a middle-aged female with no angina symptoms, who had ST elevations on electrocardiogram (EKG), which were supported by elevated cardiac enzymes and wall motion abnormalities on echocardiogram but with no culprit lesion on the coronary angiogram (CA). Optical coherence tomography (OCT) failed to reveal any critical stenosis but showed non-critical lesions in the left anterior descending artery (LAD). Percutaneous coronary intervention (PCI) of the LAD non-critical lesion with drug-eluting stent (DES) was performed due to strong suspicion for myocardial infarction and to decrease the risk for further thrombotic events as well as cardiomyopathy.

## Case presentation

**Figure 1:**
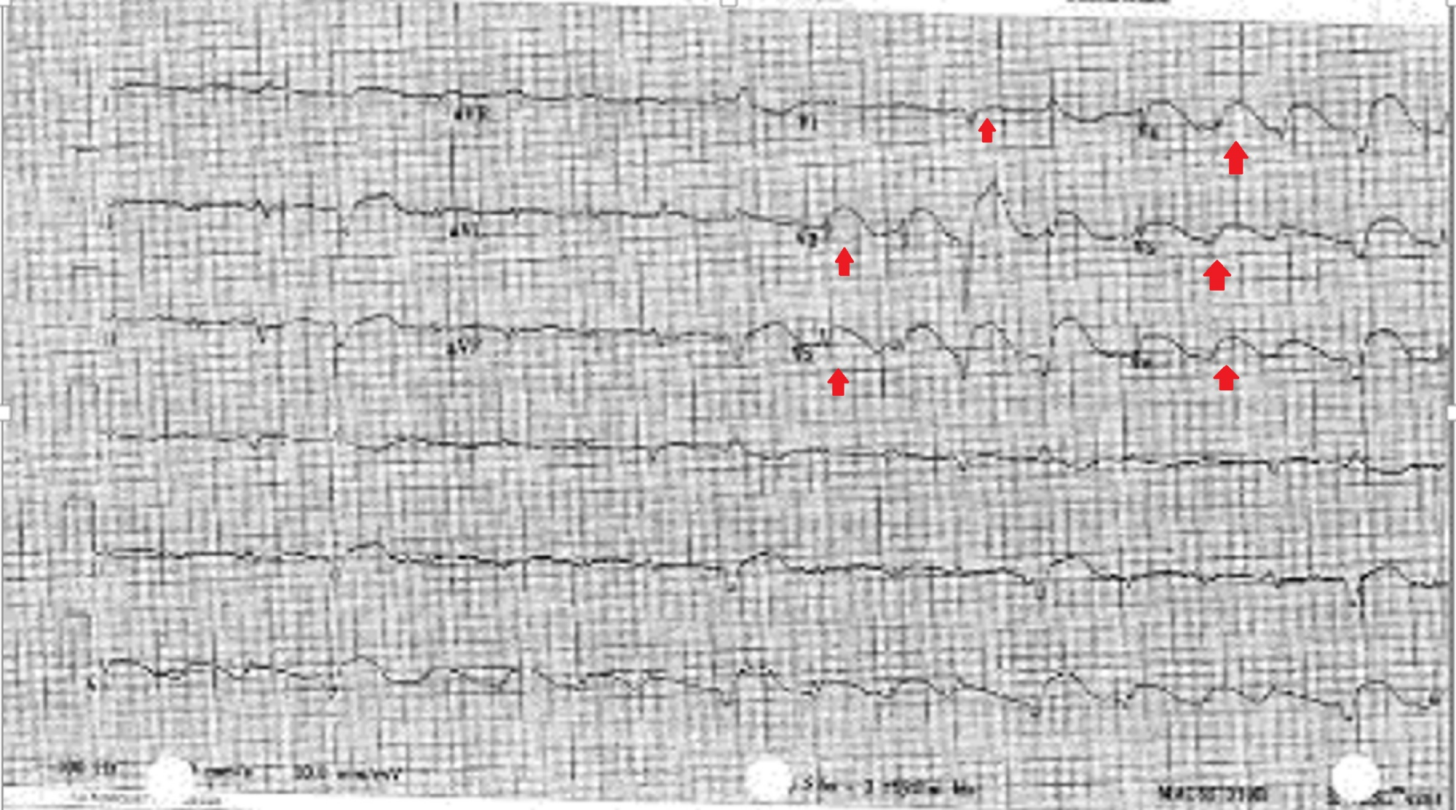
Electrocardiogram on presentation showing diffuse, convex ST-segment elevations in leads V1 to V6

A 50-year-old female presented with intractable nausea and vomiting after a hiking trip. Due to severe dehydration and metabolic acidosis, she was started on bicarbonate drip and admitted to the intensive care unit (ICU). The patient did not have any history significant for anginal chest pain and declined any symptoms of angina during current admission. Her previous history did not reveal any coronary artery disease risk factors, including hypertension, diabetes, hyperlipidemia, or smoking. Family history was also unremarkable. However, EKG upon transfer showed anterolateral ST-elevations in leads V1-V6 (Figure 1).

**Figure 2:**
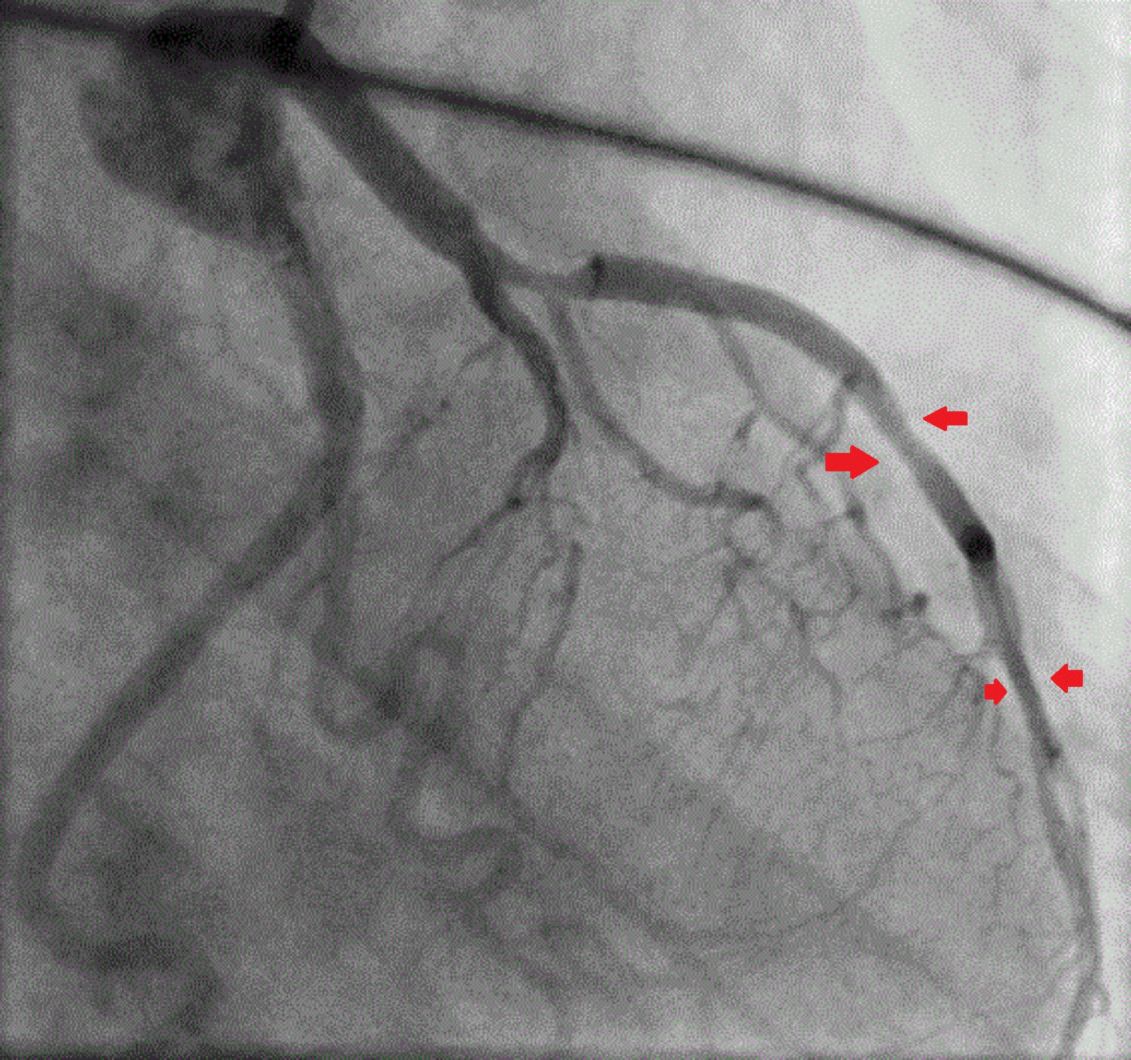
Coronary angiography showing the mid-left anterior descending artery 70% and the distal left anterior descending artery 60% lesion but with good flow in the left coronary artery system (shown by the free flow of contrast)

Cardiac troponin that was normal on admission peaked to 50 ng/ml after 15 hours of initial presentation. The patient was loaded with aspirin and clopidogrel and started on an unfractionated heparin infusion. She was referred for immediate left heart catheterization (LHC), which revealed 70% mid-segment and 60% distal segment stenosis of the LAD while the other coronary arteries were normal (Figure 2).

**Figure 3:**
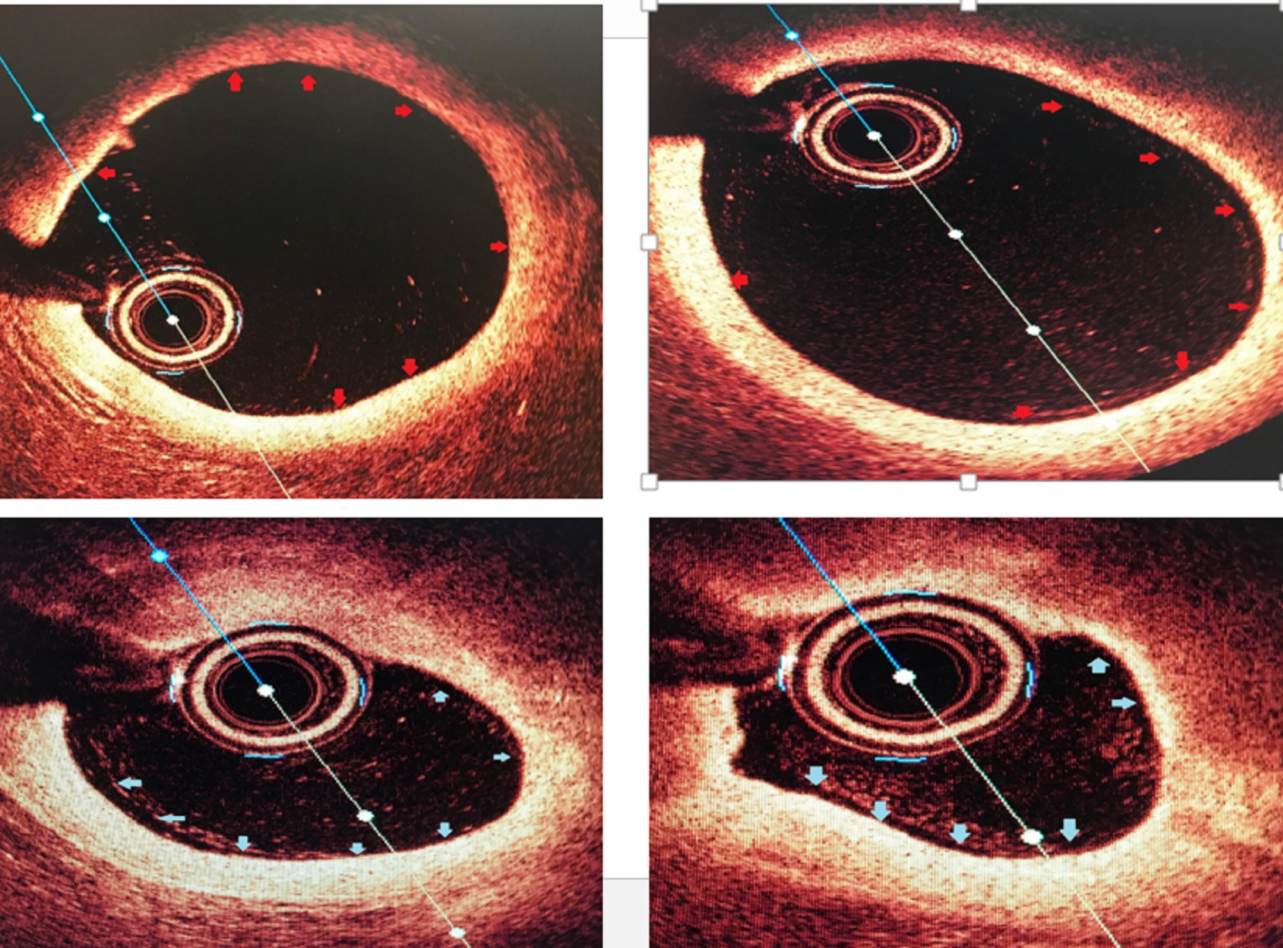
Optical coherence tomography of the left anterior descending artery (LAD) with the top left and right showing proximal LAD with minimal luminal irregularities and the bottom right and left showing mid-LAD non-critical stenosis

Intraoperative echocardiogram showed corresponding wall motion abnormalities (WMA) involving the anterolateral wall of the left ventricle. Optical coherence tomography (OCT) showed uninterrupted intima with no evidence of plaque rupture, erosion, luminal thrombus, or acute occlusion (Figure 3).

The differential diagnosis at this time included myocardial infarction with non-obstructive coronary arteries (MINOCA) vs. stress-induced cardiomyopathy vs. severe demand ischemia. Given the clinical presentation of myocardial infarction suggested by elevated cardiac enzymes and supported by WMA on echocardiogram, it was decided to treat the mid-LAD lesion with a drug-eluting stent followed by dual antiplatelet treatment (DAPT) with aspirin and clopidogrel for 12 months.

## Discussion

Acute coronary syndrome (ACS) encompasses a spectrum of unstable angina, non-ST-elevation myocardial infarction (NSTEMI), and ST-elevation myocardial infarction (STEMI). An 'unstable plaque' with a thin fibrous cap and a lipid-laden core usually is the culprit is such scenarios. An erosion or rupture of that plaque exposes the highly thrombotic material of the plaque to the circulating blood. This leads to platelet aggregation and activation as well as the activation of a clotting cascade, resulting in thrombosis [3-4]. A STEMI usually results from complete occlusion of the culprit vessel. However, this needs to be taken from the perspective of clinical presentation as well as other diagnostic and laboratory values. Various mimics of ST-elevation on electrocardiogram include coronary aneurysm, myo/pericarditis, cardiomyopathy, Brugada syndrome, aortic stenosis, aortic dissection, subarachnoidal hemorrhage, pneumonia, chronic obstructive pulmonary disease, mediastinal tumor, and peritonitis after recent abdominal surgery. A careful history and physical examination can rule out the majority of these diagnoses [5-6].

However, epicardial vessel occlusion is not the sole cause of myocardial infarction. Several series have reported angiographically normal coronary arteries in patients with STEMI. Yet, the underlying etiology and prognosis in most of these cases remain unclear [7-8]. Previous studies have found the incidence of angiographically normal arteries in acute myocardial infarction (AMI) to vary between 1.0% and 8.5% [9]. One study, specifically looking at 10-year data on STEMI patients, concluded that 5.7% of patients who presented with an initial diagnosis of STEMI had angiographically normal coronary arteries. The long-term outcomes of this group of patients were also found to be favorable in this group of patients [10].

Clinical data from clinical trials have shown that treating stable stenosis with an intervention does not decrease the incidence of future thrombotic events. One of these trials revealed that medical management provides as much protection from future coronary events as doing intervention [11-12].

However, we describe a rare instance of a patient with no history of angina and with no current angina symptoms who had ST-elevation MI on EKG, supported by elevated cardiac enzymes and echocardiography but with no culprit lesion to suggest a total occlusion of the corresponding coronary artery. Since this happened in the setting of severe dehydration, the stable lesions in the diseased LAD can be postulated to be the culprit. However, it is rare to see, even in patients who have stable coronary artery lesions and significant dehydration, a picture of STEMI such as this [13].

Current evidence for stenting the stable lesions in the absence of any anginal symptoms is weak [14-15]. However, with this rare clinical presentation, we decided to stent the lesion with DES followed by DAPT for one year, as there was significant myocardial injury evident by elevated cardiac enzymes and wall motion abnormalities, predisposing the patient to further thrombotic events as well as cardiomyopathy.

## Conclusions

STEMI in the absence of an angiographically visualized culprit lesion is rare. When guided by further intravascular imaging like OCT, an uninterrupted intima may be seen. The treatment of noncritical lesions seen on angiography and OCT in this scenario is a topic of debate. When supported by evidence for significant myocardial injury (elevated cardiac enzymes, wall motion abnormalities on echocardiogram, or ischemia/infarction on stress testing), stenting of these lesions can be performed. However, in the absence of any coronary artery lesion and angiographically normal coronary arteries, medical management with risk factor control is reasonable. 
